# Olaparib versus Placebo in Maintenance Treatment of Germline BRCA-Mutated Metastatic Pancreatic Cancer: A Cost–Utility Analysis from the Canadian Public Payer’s Perspective

**DOI:** 10.3390/curroncol30050354

**Published:** 2023-05-02

**Authors:** Fatemeh Mirzayeh Fashami, Mitchell Levine, Feng Xie, Gordon Blackhouse, Jean-Eric Tarride

**Affiliations:** 1 Health Research Methodology Graduate Program, McMaster University, 1280 Main St. West, Hamilton, ON L8S 4K1, Canada; 2 Department of Health Research Methods, Evidence, and Impact, McMaster University, 1280 Main St. West, Hamilton, ON L8S 4K1, Canada; 3 Center for Health Economics and Policy Analysis (CHEPA), McMaster University, 1280 Main St. West, Hamilton, ON L8S 4K1, Canada; 4 Programs for Assessment of Technology in Health (PATH), The Research Institute of St. Joe’s Hamilton, St. Joseph’s Healthcare Hamilton, 50 Charlton Ave E, Hamilton, ON L8N 4A6, Canada; 5 McMaster Chair in Health Technology Management, McMaster University, 1280 Main St. West, Hamilton, ON L8S 4K1, Canada

**Keywords:** olaparib, metastatic pancreatic cancer, cost–utility, public payer, Canada

## Abstract

Pancreatic cancer has an annual incidence of 2/10,000 in Canada, with a one-year mortality rate greater than 80%. In the absence of a cost-effectiveness analysis in Canada, this study’s objective was to assess the cost-effectiveness of olaparib versus a placebo in adult patients with deleterious or suspected deleterious BRCA metastatic pancreatic adenocarcinoma, who did not show any progression for at least 16 weeks with first-line platinum-based chemotherapy. A partitioned survival model with a 5-year time horizon was adopted to estimate the costs and effectiveness. All of the costs were extracted from the public payer’s available resources, effectiveness data were obtained from the POLO trial, and Canadian studies were used for utility inputs. Probabilistic sensitivity analyses and scenario analyses were performed. The total costs of olaparib and the placebo over five years were CAD 179,477 and CAD 68,569, with overall quality-adjusted life-years (QALYs) of 1.70 and 1.36, respectively. The incremental cost-effectiveness ratio (ICER) of the olaparib group compared with the placebo was CAD 329,517 per QALY. With a commonly cited willingness to pay (WTP) threshold of CAD 50,000 per QALY, the drug does not achieve acceptable cost-effectiveness mainly due to the high price of the medication and insufficient impact on the overall survival of patients with metastatic pancreatic cancer.

## 1. Introduction

Approximately 43% of Canadians are expected to be diagnosed with cancer in their lifetime [1]. Primarily because of hospital expenditures and physician care costs, the annual economic burden of cancer in Canada is increasing [2]. While pancreatic cancer represents only a small percentage of newly diagnosed cancer cases, accounting for slightly over 2% of all cases, it is responsible for a disproportionately high percentage of cancer deaths, with approximately 6% of all cancer deaths attributed to this disease [3]. Among 23 sites of malignancy, pancreatic cancer is the most lethal cancer with a poor prognosis, and it is expected to be the third cause of death in patients with cancer in Canada [1,4,5]. In addition, pancreatic cancer is among cancers with increasing incidence [6]. Pancreatic cancer is typically a silent disease in the early stages, and when patients develop symptoms, usually the disease is too advanced (stage IV) with metastatic spread [7]. Putting considerable effort into diagnosing pancreatic cancer in the early stages to elevate survival until now has not been successful. CA 19-9 used to be a screening option, but it is no longer being used due to the non-specificity and high number of false-positives [8,9]. Furthermore, metastatic pancreatic cancer is a challenging disease to treat. Even with the most effective first-line treatment regimens, such as FOLFIRINOX or gemcitabine plus nab-paclitaxel, the median survival rates are less than one year [10,11]. Furthermore, both of these regimens are associated with toxicity, which can lead to discontinuing some or all of the drugs at or before six months of treatment [10]. Specifically for FOLFIRINOX, no more than six months of chemotherapy is recommended for patients who respond to the treatment [10].

A recent study found that 7% of Canadian patients diagnosed with pancreatic cancer carry mutations in the BRCA1 and BRCA2 genes, with a higher prevalence observed in patients diagnosed at younger ages [12]. This suggests that out of the approximately 6900 new cases of pancreatic cancer diagnosed in Canada each year, around 500 patients may have these mutations [13]. It is well known that carrying deleterious germline mutations in the BRCA1 and BRCA2 genes can increase the risk of developing pancreatic cancer [14,15]. For patients with these mutations, olaparib is currently the only gene-targeted medication that is available.

Olaparib (brand name Lynparza) was developed and marketed by AstraZeneca in 2014. Olaparib is an inhibitor of the poly (ADP-ribose) polymerase (PARP) enzyme, which is involved in DNA transcription and repair. This inhibits the growth of tumor cell lines and decreases tumor growth [16]. The medicine is available in oral tablets of 100 mg and 150 mg in the US and Canada. For metastatic pancreatic cancer, olaparib is indicated for patients with a documented deleterious or suspected deleterious germline mutation in BRCA1 or BRCA2 who have received at least 16 weeks of continuous first-line platinum-based chemotherapy and showed no evidence of disease progression, and it should be administered 300 mg twice daily [17].

In Ontario, the most populated province of Canada, the annual cost by the public payer for managing pancreatic cancer in the terminal stage is CAD 54,152 per patient [18]. Although olaparib has shown improvement in progression-free survival (PFS), it is unclear whether the public payer should include this medicine in their provincial plan since no cost-effectiveness analysis has been conducted in Canada. While a few cost-effectiveness studies have been published on the use of olaparib in metastatic pancreatic cancers (MPCs) in the USA and China, no studies have assessed olaparib’s cost-effectiveness for MPC in Canada [19,20,21]. Therefore, to inform Canadian decision-makers, the study objectives were to assess the cost-effectiveness of olaparib versus a placebo in adult patients with deleterious or suspected deleterious BRCA MPC who did not show any progression for at least 16 weeks with first-line platinum-based chemotherapy.

## 2. Materials and Methods

A cost–utility analysis (CUA) was conducted with a target population of adult patients with pancreatic adenocarcinoma and a documented deleterious or suspected deleterious germline mutation in BRCA1 or BRCA2, who had received at least 16 weeks of continuous first-line platinum-based chemotherapy and showed no evidence of disease progression [22]. Adopting the perspective of the publicly funded healthcare payer in Canada, all costs and outcomes were restricted to those relevant to the publicly funded healthcare payer. Because the survival rate of MPC is only 8% at 5 years, a 5-year time horizon was adopted for the base case analysis. In the scenario analysis, a four-year time horizon was employed.

### 2.1. Model Overview

**Figure 1:**
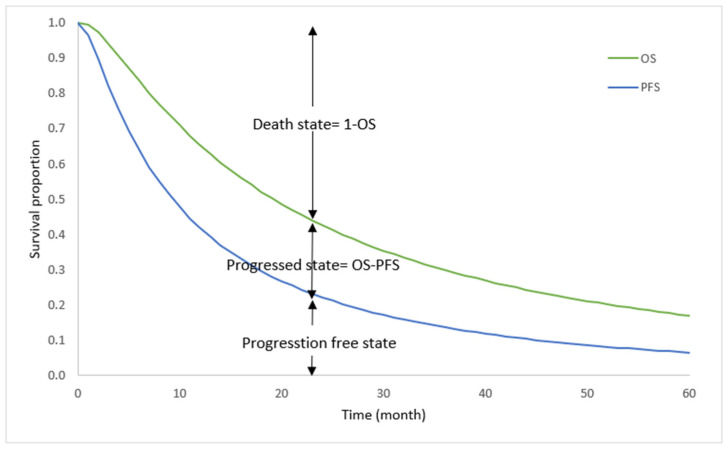
Structure of the partitioned survival model.

A partitioned survival model (PSM) including three mutually exclusive health states (progression-free, progressed, and dead) was adopted. A cohort of patients started the intervention in cycle 1 of the progression-free state. Then, in each cycle, PSM estimated the proportion of the cohort’s progression-free survival (PFS), overall survival (OS), and death based on the parametric survival functions. The population cohort of patients was followed from initiation of intervention to death or until the end of the time horizon. A one-month cycle was used for the model mainly because the follow-up tests and physician’s visits were conducted monthly. The model is demonstrated in Figure 1.

The model was assessed using an internal validation procedure involving double-checking all of the mathematical calculations and parameters. The validation of the model involved individualized validation for each parameter to see whether the results changed in the appropriate direction and amount. In the parametric survival function, the survival numbers in the OS and PFS states were calculated in R and Excel in parallel to ensure that they had the same results.

### 2.2. Clinical Input

The POLO randomized controlled trial (RCT) was used to obtain baseline characteristics, survival data points for both OS and PFS health states, adverse event rates, and severity [23]. POLO was the most comprehensive multicentral RCT conducted in 119 cities and 12 countries with 92 patients on olaparib and 62 patients on a placebo, and it was assessed to be fit for purpose and credible to be used in this study [24]. All patients in the POLO trial were followed up until they were dead or left the study. The proportion of patients who were censored from the study was the same between the two groups. Based on the results of the POLO trial, it was assumed that the rate and severity of grade 3 and 4 adverse events were not significantly different between the intervention and placebo group; hence we assumed the same and did not add adverse events to the model.

**Figure 2:**
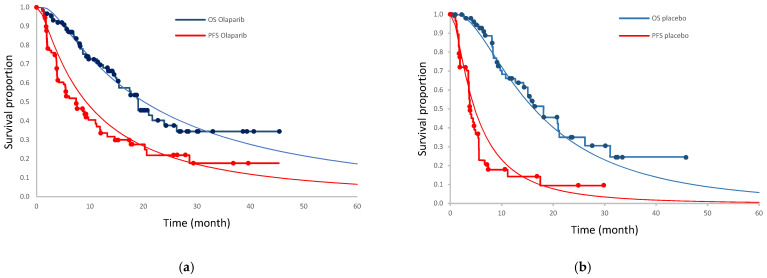
Replicated Kaplan–Meier OS and PFS survival curves along with the log-normal parametric curves for predicted PFS and OS in (**a**) olaparib group and (**b**) placebo group.

Since patient-level data were not accessible, the web-Plot Digitizer version 4.3 software was used to extract Kaplan–Meier OS and PFS data points from the POLO trial via a precise method explained by Hoyle and Henley [25,26]. After extracting the non-parametric Kaplan–Meier survival data points from the trial, R software was used to fit the parametric survival functions, including exponential, Weibull, log-normal, log-logistic, and generalized gamma. The goodness of fit of parametric models was based on the Akaike information criterion (AIC). The log-normal survival function had the lowest AIC in all of the curves except for PFS in the placebo group (Supplementary Table S1). In the PFS placebo, the RP spine (k = 2) with AIC equal to 204.30 had the least AIC value, but was not used in the base case analysis because the differences between the PFS and OS curves resulted in OS values less than progression-free values, which is not logical. Therefore, log-normal curves were adopted for all of the survival parametric functions in the base case analysis. The OS and PFS plots are demonstrated in Figure 2. By having the OS and PFS curves, the proportion of patients in a progression-free state was equal to the PFS proportion in that cycle. The proportion of patients in progressed health states was calculated by subtracting PFS from OS in each cycle. In addition, death proportion was calculated by subtracting the OS proportion from 1.

### 2.3. Health State Utility

Based on the POLO clinical trial outcomes, there were no significant differences in the health-related quality of life (HRQoL) measurements between the placebo and olaparib groups. The findings indicated that utility values relied solely on the patient’s present health state [27,28]. Therefore, based on the fitness for purpose, credibility, and consistency, utility values of different health states were obtained from a study that measured the HRQoL of progression-free and progressed states in patients with MPC in Canadian patients using EQ-5D tools [29]. The utility values are shown in Table 1.

### 2.4. Resource Use and Costs

From the public payer’s perspective, costs of the treatment, the comparator, and the necessary related resources were obtained from the literature. Since olaparib is not reimbursed in Canada, the cost of olaparib was extracted from a previously published CADTH report [30]. As reimbursement decision-making in Canada is at the provincial level, all of the related costs of physicians’ visits and laboratory test fees were obtained from the OHIP Schedule of Benefits and Fees for physician services and the Ontario schedule of benefits for laboratory services [31,32].

Costs of a single CT scan and an oncologist visit every two months were applied to all patients (Table 1). The cost incurred for conducting a single CT scan was estimated at CAD 859.58, leading to a monthly expenditure of CAD 429.8 for the monthly CT scans. The cost of CA 19-9 was not included in this study since the public payers in Canada do not cover it. Apart from that, the cost of monitoring the treatment for patients in the progression-free state who received olaparib included one complete blood count and one complete biochemistry test every month. The cost of care for patients in the progressed state was calculated by adding the cost of one complete blood count and biochemistry test, and one cycle of palliative care along with the cost of third-line chemotherapy medications every month [37,38].

It was assumed that 40% of patients in the progressed health state would transition to another chemotherapy medication following treatment with platinum-based medication and olaparib [10,39]. The following assumptions were made when determining the cost of post-progression treatment. As first-line chemotherapy, PMC can be treated with different chemotherapy regimens, including FOLFIRINOX and the combination of Gemcitabine and nab-paclitaxel. FOLFIRINOX is a more commonly used first-line therapy, and we assumed that 70% of patients received this treatment before olaparib [40,41,42,43]. According to the 2020 update from the American Society of Clinical Oncology, for patients who progressed after therapy with olaparib and had used FOLIFORINOX as the first-line therapy, Gemcitabine plus nab-paclitaxel should be used following progression. For patients who received Gemcitabine plus nab-paclitaxel as their first-line therapy, fluorouracil plus nano liposomal irinotecan or fluorouracil plus oxaliplatin were regarded as their subsequent treatment [22]. The resulting cost of subsequent chemotherapy following olaparib or a placebo was calculated based on the aforementioned explanation using an arithmetic mean method which yielded a monthly average of CAD 5942. Assuming that 40% of patients will receive this treatment following progression, a weighted cost of CAD 2377 was applied to olaparib patients while in the progressed disease health state. We assumed that patients in the progressed health state needed palliative care. Regarding palliative care, the data were obtained from Canadian studies in Ontario. According to the previous real-world data, 75% and 46% of patients in the end-of-life trajectory need hospital and home palliative care, respectively [44]. Costs of hospitalization and ER visits for patients with palliative care in Canada were reported at CAD 4558 and CAD 105 in 2012, respectively. Since MPC is a disease with a poor prognosis and patients usually have three months of survival after progression of the disease, we assumed that the mentioned costs for hospitalization were for the three months of the progressed disease, and therefore they were divided into three to be used for monthly palliative care therapy [36].

The year 2022 was considered as the base year for the analysis. Costs were provided in Canadian dollars (December 2022) and the Bank of Canada Inflation Calculator was utilized to inflate the consumer price index of all of the costs that were not obtained from the year 2022 [32].

### 2.5. Discounting

In the base case analysis, an annual discount rate of 1.5% was utilized for both costs and outcomes. Scenario analyses were conducted with discount-rate scenarios of 0% and 3%.

### 2.6. Analysis

The expected values for costs and quality-adjusted life-years (QALYs) for olaparib and the placebo were calculated via probability analysis using a Monte Carlo simulation with 5000 replications. By plotting the expected values of incremental costs, incremental QALYs, and cost per QALY by the number of replications, stability was demonstrated, and hence the number of replicas was considered to be sufficient (Figure S1). Distribution was chosen so that it reflected each input parameter’s key characteristics. Both overall and progression-free survival curves became probabilistic by having random live numbers from the log-normal distributions. Beta distribution was used for utility values of progression-free and progressed health states, and gamma distribution was used for all of the costs. For the costs of palliative care and patient care, as no data were available for the degree of uncertainty, a conservation approach was adopted, and it was assumed that the standard error of the mean was 25% of the mean value. The cost of olaparib and discount rates were assumed to be fixed. Since no correlation between data was available, no correlations were assumed. A cost–utility analysis was conducted to calculate the incremental cost-effectiveness ratios.

### 2.7. Sensitivity Analysis

Probabilistic analysis was conducted to examine each parameter’s uncertainty to assess the impact of uncertainty on the outcomes of each treatment group explicitly. The results were presented via cost-effectiveness acceptability curves.

In the POLO trial, the patient neither remained at risk of death nor at risk of disease progression in the 45th month. Thus, the non-reference analysis was performed with a 4-year time horizon to assess the impact of extrapolation beyond clinical evidence. Therefore, a sensitivity analysis was performed at a 4-year time horizon to compare the results of the QALY and the costs of CEA with the base case analysis, and calculate the proportion of incremental QALY gains from olaparib after 4 years.

To explore methodological and structural uncertainty, the results of reference case analysis were compared to scenario analyses with 0% and 3% discount rates and a 4-year time horizon.

## 3. Results

### 3.1. Base Case Results

In the progression-free state, olaparib provided 1.28 progression-free life years and 1.10 progression-free QALYs with an incremental cost of CAD 110,909 compared to the placebo, which resulted in an incremental cost–utility ratio of CAD 329,517. The results are demonstrated in Table 2. At the commonly cited willingness to pay threshold of CAD 50,000 and CAD 100,000 per QALY in Canada, the probability of olaparib being cost-effective was 0% [45].

**Figure 3:**
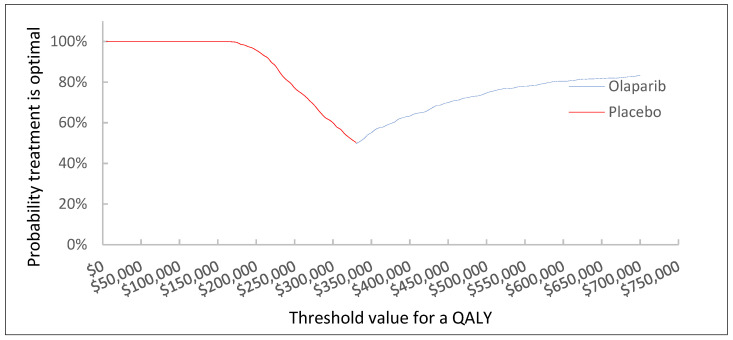
Cost-effectiveness acceptability curve.

**Figure 4:**
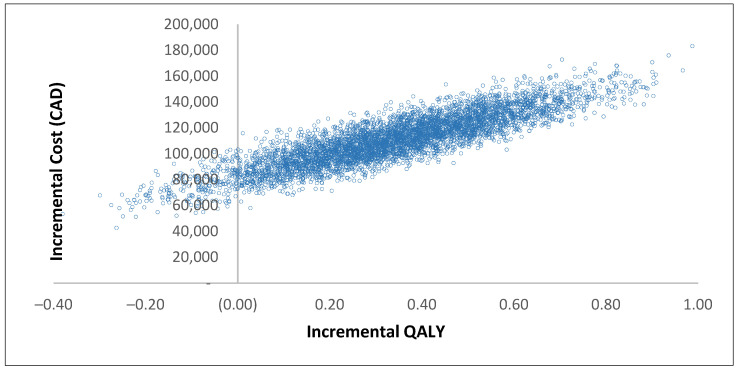
Incremental cost-effectiveness plane.

We also conducted an analysis to determine the level of price reduction required for olaparib to become cost-effective at a WTP threshold of CAD 50,000. Our results indicate that even with an 80% price reduction, the probability of olaparib being cost-effective remains at 0%. However, a price reduction of 85%, 90%, or 99% would result in probabilities of olaparib being cost-effective at 10%, 55%, or 95%, respectively. Cost-effectiveness acceptability curve and incremental cost-effectiveness plane are shown in Figure 3 and Figure 4, respectively.

### 3.2. Results of Scenario Analyses

Scenario analyses were conducted at 0% and 3% discount rates and a 4-year time horizon. At the discount rate of 0%, olaparib produced a 1.66 QALY by consuming CAD 182,912 and the placebo produced a 1.31 QALY at the cost of CAD 69,973. The ICUR for olaparib compared to the placebo at a 0% discount was CAD 322,143. At a 3% discount rate, the QALY produced by olaparib and the placebo were 1.67 and 1.34, and the related costs were CAD 176,482 and CAD 67,582, respectively. Therefore, at a discount rate of 3%, the ICUR was CAD 339,229.

By using a 4-year time horizon, olaparib and the placebo had a 1.56 and 1.31 QALY at the costs of CAD 165,353 and CAD 64,954 and produced an ICUR of CAD 405,348. The scenario analysis with a 4-year time horizon demonstrated that the proportion of the QALY gained from olaparib compared to the placebo in the 5th year was 26%. Therefore, this model indicates that a considerable proportion of QALY gains occurs after the 4th year, which the clinical trial cannot provide data for. Hence, the decision-makers might consider the uncertainty related to the time horizon. The results of the scenario analysis are demonstrated in Table 3.

Despite the changes in the outcomes in the scenario analyses, no considerable change was observed in the willingness to pay of CAD 50,000, as in all of the non-reference case analyses the ICURs were considerably more than CAD 500,000.

## 4. Discussion

Despite being approved by Health Canada and exhibiting potential benefits in improving progression-free survival (PFS) in MPC, the cost of olaparib may serve as an obstacle for the Canadian public payer to incorporate it into a reimbursement plan, thereby hindering patients and physicians from accessing this medication [46]. This study addressed the absence of a cost–utility assessment of olaparib use for MPC in Canada. Based on the clinical outcomes of the POLO trial, our study showed that the treatment of MPC with a BRCA1 or BRCA2 mutation could be optimal for WTP thresholds of more than CAD 329,517 per additional QALY in Canada.

According to the evaluation conducted by the Canadian Agency for Drugs and Technologies in Health (CADTH), olaparib has been recommended to undergo a considerable reduction in cost for first-line therapy in ovarian cancer, while its application as a second-line treatment for ovarian cancer has been rejected. Additionally, CADTH has requested a substantial price reduction of 71% for olaparib to be deemed cost-effective for the treatment of prostate cancer at a WTP threshold of CAD 50,000 [47,48,49]. However, olaparib is currently available under the Exceptional Access Program (EAP) in Ontario and is government-funded for the treatment of ovarian cancer [50]. In MPC, olaparib was shown to improve the PFS, but it did not have any effect on the OS, and this could be the main limitation for it to show cost-effectiveness in Canada since it has a marginal effect on life years gained.

Similar studies have been conducted in other jurisdictions. Wu et al. conducted a study from the US payer’s perspective and used a 1-week cycle with a 5-year time horizon [19]. Zhan et al. used a 1-month cycle and 5-year time horizon and took the perspective of Chinese society [20]. The two studies concluded that the medicine is not cost-effective for MPC from the public payer’s perspective in the US and the societal perspective in China, respectively [19,20]. In a study by Li and colleagues, the analysis was from the perspective of China and the USA healthcare system with a 21-day cycle and 5-year time horizon considered [21]. The results showed that olaparib produced 13.99 QALYs while the placebo produced 5.22 QALYs. However, this result lacks face validity since a QALY has a value between 0 and 1 and the maximum possible QALYs in a 5-year time horizon are 5. QALYs combine quantity of life with HRQoL. Quality of life (QoL) is represented by utilities, which are expressed on a 0 (death) to 1 (perfect health). Over one year, the maximum number of QALY is 1 (1 year of life with a utility of 1), and therefore, over 5 years, the maximum number of QALYs are 5. Therefore, the conclusion that olaparib is a cost-effective approach from the perspective of public payers in the US and China is not supported by the QALY values obtained in this study [21].

Our research has several limitations. Firstly, our study primarily relied on the POLO trial, which utilized a placebo as the comparator. However, for health technology assessments, it is recommended to utilize the current standard therapy as a comparator. Olaparib is approved for administration in suspected deleterious BRCA MPC patients who have undergone 16 weeks of platinum-based therapy and demonstrated no signs of progression. Following 16 months of platinum-based therapy, oncologists may opt to discontinue the treatment, continue with platinum-based therapy or transition to olaparib. It is unclear how many physicians discontinue therapy for their patients after 16 months of platinum-based therapy. Nevertheless, in the case of continuing chemotherapy, according to the MPC guideline, these patients can either continue receiving platinum-based therapy or use olaparib as the drug specific to BRCA mutations. Consequently, if enough clinical data were available, it would have been more appropriate to use platinum-based medication as another comparator. Another limitation of our study is that the POLO trial, which we mainly relied on, was a phase III trial and was not reflective of real-world clinical practice. While the POLO trial was a well-designed, large-scale study, it is important to note that maintenance olaparib was not a standard treatment option for pancreatic cancer prior to this trial. Therefore, the results observed in the POLO trial may not be directly applicable to real-world clinical settings. Furthermore, our study was limited by the fact that we only had access to secondary data from the POLO trial. Therefore, we could not conduct a net benefit framework to investigate the variability of the cost-effectiveness of olaparib in relation to patient characteristics or disease severity using patient-level data. This highlights the need for future research to incorporate patient-level data in economic evaluations of olaparib in metastatic pancreatic cancer. In addition, another limitation of this study is that we did not have access to patient-level data on treatment and healthcare resource use following disease progression. As a result, we had to make some assumptions about the costs and resource utilization following disease progression. This limitation may have introduced some uncertainty into our cost estimates. However, we made every effort to utilize the best available data and models to estimate the costs and resource utilization associated with each disease stage, which helped to mitigate this limitation’s impact on our overall conclusions.

## 5. Conclusions

Olaparib maintenance therapy in patients with metastatic pancreatic cancer and a germline BRCA mutation was assessed as not being a cost-effective treatment from the perspective of the Canadian public payer, using a WTP threshold of CAD 50,000 per QALY.

## Figures and Tables

**Table 1:** Input parameters.

Parameters	Base Estimate	Probability Distribution	Source
Monthly costs of progression-free health state in olaparib group			
Olaparib, 150 mg, 2 tablets, b.i.d ^1^	CAD 7906.8	Fixed	[30]
Consultation by medical oncologist ^2^	CAD 87.5	Gamma (16, 4.91)	[31]
Biochemistry test	CAD 121.0	Gamma (16, 7.56)	[32]
Complete blood count	CAD 3.9	Gamma (16, 0.25)	[32]
CT scan	CAD 429.9	Gamma (16, 26.86)	[33]
Monthly costs of progression-free health state in placebo group			
Consultation by medical oncologist	CAD 87.5	Gamma (16, 4.91)	[31]
CT scan	CAD 429.9	Gamma (16, 26.86)	[33]
Monthly costs of progressed health state in olaparib group			
Third-line therapy	CAD 2377	Gamma (16, 148.56)	[34,35]
Biochemistry test	CAD 121.0	Gamma (16, 7.56)	[32]
Complete blood count	CAD 3.9	Gamma (16, 0.25)	[32]
Consultation by medical oncologist	CAD 87.5	Gamma (16, 4.91)	[31]
CT scan	CAD 429.9	Gamma (16, 26.86)	[33]
ER visit	CAD 43.3	Gamma (16, 2.71)	[36]
Palliative care	CAD 1890.0	Gamma (16, 118.13)	[36]
Monthly costs of progressed health state in placebo group			
Third-line therapy	CAD 2377	Gamma (16, 148.56)	[34,35]
Biochemistry test	CAD 121.0	Gamma (16, 7.56)	[32]
Complete blood count	CAD 3.9	Gamma (16, 0.25)	[32]
Consultation by medical oncologist	CAD 87.5	Gamma (16, 4.91)	[31]
CT scan	CAD 429.9	Gamma (16, 26.86)	[33]
ER visit	CAD 43.3	Gamma (16, 2.71)	[36]
Palliative care	CAD 1890.0	Gamma (16, 118.13)	[36]
Utility values			
Progression-free state	0.81	Beta (4.73, 1.11)	[29]
Progressed state	0.73	Beta (3.71, 1.37)	[29]

^1^ b.i.d. = twice daily; ^2^ for event costs where no measures of dispersion were available, a coefficient of variation of 25% was assumed.

**Table 2:** Expected values of key outcomes for the reference case analysis.

Outcome	Olaparib	Placebo	Incremental
Cost of progression-free state	CAD 131,586	CAD 3727	CAD 127,859
Cost of progressed state	CAD 47,891	CAD 64,842	-CAD 16,951
Total cost	CAD 179,477	CAD 68,569	CAD 110,909
Progression-free life years	1.28	0.61	0.67
Progressed life years	0.81	1.09	−0.28
Total life years	2.09	1.70	0.39
QALY of progression-free state	1.11	0.56	0.53
QALY of progressed state	0.59	0.80	−0.21
Total QALY	1.70	1.36	0.34
ICUR	CAD 329,517		

ICUR: incremental cost/utility ratio, NMB: net monetary benefit.

**Table 3:** Expected values of non-reference case analysis.

Scenario	Outcome	Olaparib	Placebo	Incremental	ICUR ^1^
Base case	Cost	CAD 179,477	CAD 68,569	CAD 110,909	CAD 329,517
	QALY	1.70	1.36	0.34	
Discount rate of 0%	Cost	CAD 182,912	CAD 69,973	CAD 112,940	CAD 322,143
	QALY	1.66	1.31	0.35	
Discount rate of 3%	Cost	CAD 176,482	CAD 67,582	CAD 108,900	CAD 339,229
	QALY	1.67	1.34	0.32	
4-year time horizon	Cost	CAD 165,353	CAD 64,954	CAD 100,339	CAD 405,348
	QALY	1.56	1.31	0.25	

^1^ ICUR: incremental cost/utility ratio.

## Data Availability

The data presented in this study are available on request from the corresponding author.

## Supplementary Materials

The following supporting information can be downloaded at https://www.mdpi.com/article/10.3390/curroncol30050354/s1: Table S1. Estimated parameters for each survival model; Figure S1. Impact of number of simulations on expected values.
