# Supplementary material for: Olaparib versus Placebo in Maintenance Treatment of Germline BRCA-Mutated Metastatic Pancreatic Cancer: A Cost–Utility Analysis from the Canadian Public Payer’s Perspective
Source: Curr Oncol. 2023 May 2;30(5):4688–99. doi: 10.3390/curroncol30050354 (PMC10217075; doi:10.3390/curroncol30050354)
Supplement: Supplementary file 1 [file curroncol-30-00354-s001.zip › curroncol-2290206-supplementary.pdf]

Supplementary appendix

Table S1.: Estimated parameters for each survival model.

		Est	L95%	U95%	SE	AIC
**OS Placebo**						
Exponential		0.040	0.029	0.055	0.007	305.70
Weibull	Shape	1.488	1.158	1.913	0.191	299.80
	scale	22.638	18.140	28.252	2.559	
Log normal	meanlog	2.790	2.550	3.029	0.122	293.82
	sdlog	0.830	0.655	1.052	0.101	
Log-logistic	Shape	2.056	1.580	2.675	0.276	295.15
	scale	16.043	12.668	20.317	1.933	
generalized gamma	Shape	0.255	0.089	0.733	0.137	296.56
	scale	0.000	0.000	105,719.400	0.001	
	k	24.184	3.090	189.251	25.386	
RP spline K=0	gamma0	- 4.643	- 5.811	- 3.475	0.596	299.80
	gamma1	1.488	1.114	1.862	0.191	
RP spline K=1	gamma0	- 7.346	- 10.207	- 4.485	1.460	295.33
	gamma1	2.954	1.561	4.348	0.711	
	gamma2	0.454	0.073	0.836	0.195	
RP spline K=2	gamma0	- 7.512	-11.345	- 3.679	1.956	297.41
	gamma1	3.067	0.924	5.210	1.094	
	gamma2	0.477	- 1.654	2.609	1.087	
	gamma3	- 0.002	- 2.117	2.114	1.079	
**OS olaparib**						
exponential		0.034	0.026	0.046	0.005	377.80
Weibull	Shape	1.180	0.923	1.508	0.148	378.19
	scale	27.116	20.741	35.451	3.708	
Log normal	meanlog	2.952	2.640	3.265	0.159	375.53
	sdlog	1.194	0.955	1.493	0.136	
Log-logistic	Shape	1.490	1.160	1.913	0.190	375.57
	scale	18.810	14.149	25.006	2.733	
generalized gamma	Shape	0.258	0.033	2.010	0.270	377.60
	scale	0.001	0.000	1469654000000	0.022	
	k	12.357	0.286	534.784	23.753	
RP spline K=0	gamma0	-3.893	-4.796	-2.990	0.461	378.19
	gamma1	1.180	0.890	1.469	0.148	
RP spline K=1	gamma0	-4.583	-6.120	-3.046	0.784	378.56
	gamma1	1.670	0.787	2.552	0.450	
	gamma2	0.109	-0.067	0.286	0.090	
RP spline K=2	gamma0	-4.451	-6.003	-2.899	0.792	380.2
	gamma1	1.500	0.458	2.541	0.531	
	gamma2	-0.149	-0.913	0.615	0.390	
	gamma3	0.376	-0.759	1.511	0.579	

**PFS placebo**						
exponential		0.132	0.094	0.183	0.022	213.96
Weibull	Shape	1.044	0.817	1.334	0.131	215.84
	scale	7.624	5.546	10.480	1.238	
Log normal	meanlog	1.558	1.253	1.864	0.156	206.29
	sdlog	1.012	0.794	1.289	0.125	
Log-logistic	Shape	1.756	1.328	2.324	0.251	205.58
	scale	4.582	3.435	6.112	0.674	
generalized gamma	Shape	0.180	0.038	0.853	0.143	209.38
	scale	0.000	0.000	5264922000000	0.000	
	k	31.302	1.434	683.318	49.242	
RP spline K=0	gamma0	-2.120	-2.756	-1.484	0.325	215.84
	gamma1	1.044	0.788	1.300	0.131	
RP spline K=1	gamma0	-3.146	-4.223	-2.069	0.549	206.31
	gamma1	2.468	1.432	3.503	0.528	
	gamma2	0.251	0.091	0.410	0.081	
RP spline K=2	gamma0	-2.922	-3.899	-1.945	0.499	204.30
	gamma1	1.549	0.426	2.672	0.573	
	gamma2	-1.464	-2.999	0.072	0.783	
	gamma3	1.673	0.165	3.181	0.770	
**PFS Olaparib**						
exponential		0.1316	0.0646	0.0484	0.0862	0.009
Weibull	Shape	0.965	0.770	1.209	0.111	347.97
	scale	15.520	11.497	20.950	2.376	
Log normal	meanlog	2.228	1.910	2.547	0.162	340.00
	sdlog	1.235	0.999	1.526	0.134	
Log-logistic	Shape	1.356	1.071	1.715	0.163	342.42
	scale	9.098	6.592	12.557	1.496	
generalized gamma	Shape	0.167	0.031	0.891	0.143	343.12
	scale	0.000	0.000	1414691000000000	0.000	
	k	24.680	0.944	645.449	41.099	
RP spline K=0	gamma0	-2.644	-3.293	-1.995	0.331	347.97
	gamma1	0.964	0.747	1.182	0.111	
RP spline K=1	gamma0	-3.396	-4.373	-2.418	0.499	342.27
	gamma1	1.693	1.049	2.337	0.329	
	gamma2	0.108	0.028	0.189	0.041	
RP spline K=2	gamma0	-3.633	-4.784	-2.481	0.587	343.18
	gamma1	2.107	1.019	3.195	0.555	
	gamma2	0.344	-0.189	0.878	0.272	
	gamma3	-0.186	-0.630	0.259	0.227	

Abbreviations:

AIC: Akaike information criterion; Est: point estimation; L95%: lower boundary of 95% confidence interval; U95%: upper boundary of 95% confidence interval.

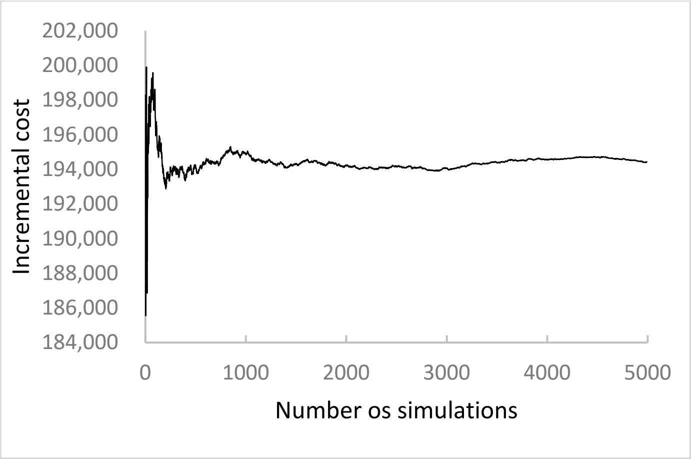

p. Incremental costs of olaparib versus placebo

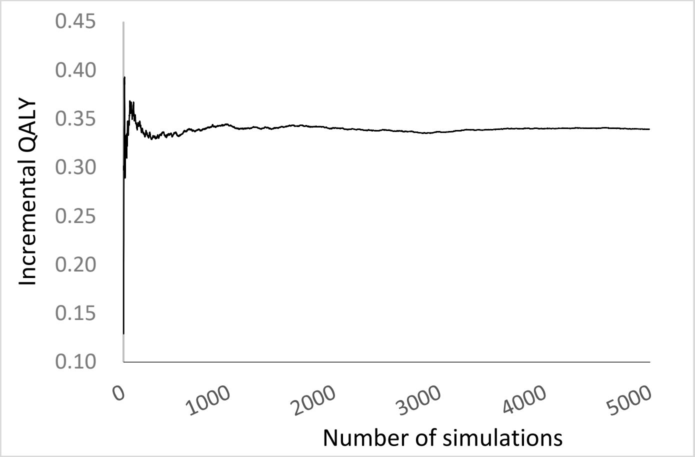

q. Incremental QALYs of olaparib versus placebo

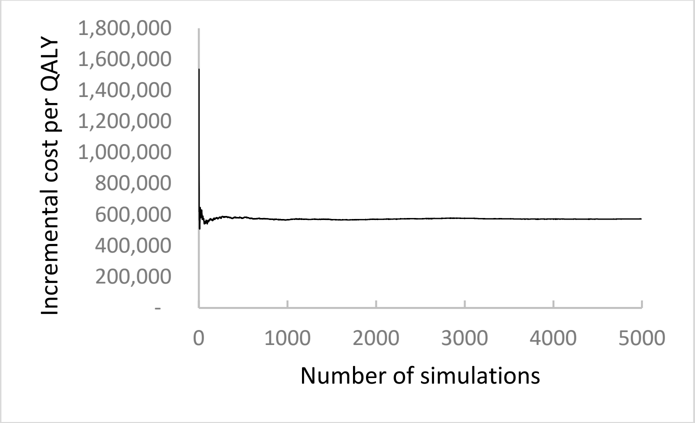

q. Incremental cost per QALY gained of olaparib versus placebo

Figure S1. Impact of number of simulations on expected values.
